# Health-Promoting Behaviors and Their Association With Certain Demographic Characteristics of Nursing Students of Tehran City in 2013

**DOI:** 10.5539/gjhs.v7n2p264

**Published:** 2014-11-16

**Authors:** Meimanat Hosseini, Tahereh Ashktorab, Mohammad HosseinTaghdisi, Ali Esmaeili Vardanjani, Hossein Rafiei

**Affiliations:** 1 Department of Community Health Nursing, Faculty of Nursing and Midwifery, Shahid Beheshti University of Medical Sciences, Tehran, Iran; 2 Department of Medical-Surgical, Faculty of Nursing and Midwifery, Shahid Beheshti University of Medical Sciences, Tehran, Iran; 3 Department of Health Promotion, Faculty of Tehran University of Medical Sciences, Tehran, Iran; 4 Chronic Disease (home care) Research Center, Hamadan University of Medical Sciences, Hamadan, Iran; 5 Department of Medical Surgical Nursing, School of Nursing and Midwifery, Shahrekord University of Medical Sciences, Shahrekord, Iran

**Keywords:** health-promoting behavior, demographic characteristics, student, nursing

## Abstract

**Background and Objectives:**

Nursing students provide health services and their behavior may affect the clinical services that they provide to clients. In addition, Health-promoting behaviors among nursing students may affect the quality of patient education. Therefore, the aim of this study was to determine the relationship between health-promoting behaviors and certain demographic characteristics of nursing students in Tehran.

**Methods:**

This cross-sectional study was performed on 404 undergraduate nursing students (107 males and 297 females) of universities of Tehran who were selected through stratified random sampling procedure. The data collection tools were: demographic characteristics questionnaire and health-promoting lifestyle profile 2. This standard instrument has 6 subscales of nutrition, physical activity, spiritual growth, health responsibility, interpersonal relations, and stress management. In order to keep the weight of the expressions of the tool and subscale scores the mean were calculated. For this purpose, each expression was scored on a scale of 1 to 4. Internal consistency (Cronbach’s Alpha) of this tool was a=0.86. Data was analyzed by using statistical tests of Chi Square, Independent T and ANOVA.

**Results:**

According to the results, health-promoting behaviors among the students scored highly. The analysis of subscales of health-promoting lifestyle profile 2 showed that the highest mean belongs to the subscale of spiritual growth and the lowest mean belongs to the subscale of physical activity. The mean of health-promoting behaviors among married students was higher than among single students. Moreover, as students advanced through academic years, health responsibility increased and stress management reduced.

**Discussion:**

Undoubtedly, physical activity and stress management have a significant role in personal health and occupational satisfaction of students, which consequently help them provide better services to clients. Therefore, considering the low scores of students in these sections, nursing education managers, policymakers and planners should pay attention to these problems.

## 1. Introduction

In view of the increased costs of healthcare and the fact that patient care is not the only health factor, there has been an increased support for health promotion concept and its extension among healthcare personnel, particularly nurses. Health-promoting activities and healthy lifestyle have been demonstrated to be efficient healthcare strategies (Haddad et al., 2004). If people choose a lifestyle in accordance with economic prosperity and interpersonal balance, both individuals and the community would enjoy the general benefits of health promotion. Furthermore, health promotion will reduce healthcare costs in future (Peterson & Bredow, 2004).

Health-promoting behavior is an important concept in nursing, because most of nursing activities involve healthcare education (Chhim, 2011). In nursing, health promotion should be considered as healthy nursing or interactive health-promoting method (Liimatainen, 2001). In this matter, American Nurses Association Code of Ethics (2001) explicitly points out that nurses are responsible for the health of both themselves and others. Likewise (Hensel, 2011), National Ethics Code of Iran Nursing Organization (2011) points out that nurses should make effort in line with public health promotion, prevention of diseases, and alleviation of pain and suffering of the public (Information Center of Iran Medical Council, 2011).

As future healthcare personnel, nursing students not only have potential effect on public health but also can possibly play an important role in healthcare domain. Therefore, health protection and promotion among nursing students is one of the key factors in universities (Wittayapun et al., 2010).

Pender suggests that healthy lifestyle behaviors are: spiritual growth, health responsibility, exercise, nutrition, interpersonal support, and stress management (Eshah, 2011), which are assessable by Health-Promoting Lifestyle Profile 2. This tool assesses health-protecting behaviors, which reduce the risk of disease, and health-promoting behaviors including welfare, self-esteem and personal works (Walker and Hill-Polerecky, 1996). Furthermore, many studies have demonstrated that there is a significant relationship between social-demographic variables such as age, education, income, and health status perception and subscales of Health-Promoting Lifestyle Profile (Eshah, 2011).

Nursing education process may affect health-promoting behaviors. Some studies showed that nursing education improves some aspects of lifestyle (Irazusta et al., 2006). On the other hand, some studies indicate that nursing education results in increased cigarette smoking and reduced welfare of students (Hensel, 2011). In this connection, Nursing Students Association of Canada (2009) has conducted a research on health-promoting behaviors of nursing students and found that physical activity of fourth year students have considerably decreased compared to first year students and that there is no difference between first year students and fourth year students in terms of stress management. In a study on nutritional status of nursing students, 23% of students reported that they rarely eat well. 69% of students said they drink only two glasses of water or other liquid in clinical work days (Canadian Nursing Students’ Association, 2009).

The subject of health valuation by nurses has remained unclear. Some feel that nurses are people with dependent character who are identified by the role of healthcare service provision and consequently lose their self-esteem. Some studies show that nurses do not perform necessary activities for a healthy lifestyle, which may affect their performance as providers of healthcare service. Considering that nursing faculties educate nurses on basics of healthy lifestyle, the question is why such behaviors are not seen among nursing students. The problem is that there is not sufficient knowledge on the factors influencing nurses’ decision to have a healthy life. Therefore, studies on health promotion needs among students may help them adopt health-promoting behaviors in their life (Jackson et al., 2007). The aim of this research is to determine the relationship between health-promoting behaviors and certain demographic characteristics of nursing students in Tehran.

## 2. Method

This cross-sectional descriptive study was performed on 404 students (107 males and 297 females) of universities of Tehran who were selected through stratified random sampling procedure. Sampling criteria were as follows: 1) participants are undergraduate nursing students in one of the universities of Tehran; 2) they were Iranian; 3) they agreed to participate in the study; and 4) they were healthy.

Data collection tools were demographic characteristics questionnaire consisting of 14 items and health-promoting lifestyle profile 2 consisting of 52 items. This standard instrument has 6 subscales of nutrition, physical activity, intellectual growth, health responsibility, interpersonal relationship, and stress management. Each option is given a point from 1 to 4. Items are “1 = Never”, “2 = Sometimes, 3 = Often, and 4 = Always. Total point is 52 in minimum and 208 in maximum. Total point of all scales was achieved by calculating the mean of answers to 52 items and the point of 6 subscales was achieved by calculating the mean of answers to the items of subscales. In order to keep the weight of expressions of the tool and make a targeted comparison between scores of subscales, the mean were calculated. Since Health-Promoting Lifestyle Profile 2 has been psychometrically evaluated, only internal consistency was determined in this study, which was a=0.86. Psychometric evaluation of this tool has been performed by determination of validity (face validity, content validity, and construct validity) and reliability (internal and external consistency). Instrument content validity index indicates minimum score of 70.84 and maximum score of 1. Construct validity by confirmatory factor analysis method has shown that this instrument has 6 subscales. The reliability of instrument was determined using internal consistency (Cronbach’s Alpha) and external consistency (Intra Class Correlation (ICC) and Wilcoxson Test), (Hosseini et al., 2010).

Upon coordination with managers of universities of medical sciences and educational deputies of the Faculty of Nursing and Midwifery of Shahid Beheshti University of Medical Sciences in Tehran, Iran, and Shahed and Baqiatollah Universities, and upon obtainment of informed consents of the participants, the research tools were distributed among the students to be completed. The needed time for completing the tools was calculated to be 15-20 minutes. For the purpose of sampling, one of the researchers obtained from education department of the faculties the list of hospitals, classes and number of undergraduate nursing students in each hospital ward and each class. Then the classes and hospitals were selected randomly. Data was analyzed by SPSS, version 20. Mean, standard deviation and percentage were used in descriptive statistics and Chi Square test, Independent T test and variance analysis were used in inferential statistics.

## 3. Findings

The results showed that age, Height, weight, body mass index and the number of family members of female students are less than male students (Table 1).

**Table 1:** Demographic and anthropometric characteristics of nursing students under study, separated by gender, in the city of Tehran in 2012-2013

Variable	Female (n = 297)		Male (n = 107)	

	Mean	Standard Deviation	Mean	Standard Deviation
Age	21.10	2.20	21.80	2.67
Height (centimeter)	163.47	5.42	177.22	6.16
Weight (Kg)	58.15	9.17	73.95	10.72
Body mass index	21.80	3.15	23.54	6.16
Number of family members	4.82	1.58	5.25	1.62

According to the results, 27.9% of female students were second year nursing student and 34.6% of male students were first year nursing student. 88.8% of female students and 90.6% of male students were single. 65.5% of female students and 52.8% of male students were of Fars race. Body mass index of 74.4% of female students and 61.7% of male students was in healthy and normal range. Chi Square test showed that there is a statistically significant relationship between gender of students and body mass index (p<0.05). 70% of female students lived with their family members and 50.5% of male students did not live with their family members. Chi Square test also showed that there is a statistically significant relationship between gender of students and whether or not they live with their family members (p<0.05). (Table 2)

**Table 2:** Frequency distribution of demographic characteristics of nursing students under study, separated by gender, in the city of Tehran in 2012-2013

Variable	Variable Classes	Female (n = 297)		Male (n = 107)	

		Percentage	Number	Percentage	Number
	1	21.5	64	34.6	37
Study year	2	27.9	83	16.8	18
	3	23.9	71	28	30
	4	26.6	79	20.6	22
Marital status	Single	88.8	261	90.6	96
	Married	11.2	36	9.4	11
	Fars	65.5	192	52.8	56
Race	Turk	16.4	48	22.6	24
	Lor	4.4	13	6.5	7
	Other	6.5	19	6.5	7
	Low	11.1	33	4.7	5
	Healthy (normal)	74.4	221	61.7	66
Body mass index	Extra weight	10.4	31	31.8	34
	Fat	2.7	8	1.9	2
Residence type	Living wih family members	70	208	49.5	53
	Not living with family members	30	89	50.5	54

According to results, the mean of female students in subscales of nutrition, interpersonal relationship, health responsibility, spiritual growth, stress management, and total scale of health-promoting lifestyle profile 2 was higher than male students. In subscale of physical activity, the mean of male students was higher than females. Independent T test revealed that there is a statistically significant difference between male and female students in subscales of nutrition, physical activity, interpersonal relations, and health responsibility (p<0.05). (Table 3)

**Table 3:** Mean, standard deviation and significance level of health-promoting lifestyle profile 2 and its subscales, separated by gender and total number of nursing students

Subscale	Range	Female (n=297)	Male (n=107)	Total (n=404)	P value
Nutrition	1-4	2.63±0.41	2.48±0.47	2.59±0.43	0.002
Physical activity	1-4	1.96±0.51	2.23±0.62	2.03±0.55	0.001
Interpersonal relationship	1-4	2.99±0.39	2.80±0.42	2.94±0.41	0.001
Health responsibility	1-4	2.43±0.49	2.31±0.48	2.40±0.49	0.03
Spiritual growth	1-4	2.90±0.52	2.83±0.55	2.88±0.53	0.226
Stress management	1-4	2.42±0.43	2.35±0.50	2.40±0.45	0.178
Total behaviors	1-4	2.57±0.34	2.51±0.37	2.55±0.35	0.116

In order to determine the status of health-promoting behaviors and the associated factors, scores of students were divided into four equal parts and thereby score quarters were determined. According to study results, 53.7% of students perceived health-promoting behaviors at high level. Nutrition, physical activity, health responsibility, and stress management were perceived by 52.5%, 51.5%, 53.7%, and 50.7% of students respectively at fair level. 62.9% of students perceived interpersonal relations and 51.5% perceived spiritual growth at high level.

According to the results, the mean of health-promoting behaviors among married students was higher than single students. The mean of subscales of physical activity and stress management in single students was higher than married students. In other subscales, married students had higher scores than single students. The difference between single and married students in subscales of nutrition and spiritual growth was significant (p<0.05).

As regards the year of study and health-promoting behaviors, the results indicated that the mean of first and third year students was higher than students of other years. The mean of subscales of nutrition, physical activity, and stress management in first year students was higher than students of other years. Fourth year students perceived health responsibility more than other groups did. Variance analysis test indicated that there is a significant relationship between the groups in terms of health responsibility and stress management. Tukey test showed that there is a significant difference between first year students and other groups in stress management, and there is a significant difference between fourth year students and other groups in health responsibility (P<0.05). Fourth year students had the lowest scores in stress management (Table 4).

**Table 4:** Mean, standard deviation and significance level of subscales of health-promoting lifestyle, separated by year of study of nursing students

Subscale	First Year (n=101)	Second Year (n=101)	Third Year (n=101)	Fourth Year (n=101)	P value
Nutrition	2.65±0.45	2.53±0.42	2.60±0.41	2.59±0.45	0.281
Physical activity	2.10±0.61	1.94±0.53	2.05±0.56	2.04±0.49	0.197
Interpersonal relationship	2.91±0.46	2.94±0.39	3.01±0.41	2.91±0.41	0.259
Health responsibility	2.30±0.51	2.37±0.46	2.40±0.44	2.51±0.52	0.026
Spiritual growth	2.93±0.59	2.83±0.49	2.96±0.47	2.81±0.56	0.124
Stress management	2.49±0.51	2.40±0.39	2.39±0.43	2.31±0.46	0.045
Total behaviors	2.59±0.44	2.51±0.33	2.59±0.34	2.54±0.35	0.328

With respect to body mass index, the results indicated that the mean of nutrition, interpersonal relationship, health responsibility, stress management, and total health-promoting behaviors in low body mass index group was higher than other groups. The mean of spiritual growth in healthy body mass index group was higher than other groups and the mean of physical activity in fat group was more than other groups. Tukey test indicated that there is a significant difference between fat students and other groups in subscale of spiritual growth and there is a significant difference between low body mass index group and other groups in subscale of interpersonal relationship (p<0.05).

With respect to residence type, findings revealed that the mean of physical activity, stress management, nutrition, and health-promoting behaviors in the group who did not live with their family members was higher than the groups who lived with their family members. The mean of interpersonal relationship, health responsibility and spiritual growth in the group who lived with their family members was higher than the groups who did not live with their family members. Tukey test indicated that there is a significant difference between the students who lived with their family members and other group in subscale of nutrition (p<0.05). In other subscales, there was no significant difference between the two groups in terms of residence type.

## 4. Discussion

This study analyzed health-promoting behaviors among undergraduate nursing students in the city of Tehran. According to study results, mean and standard deviation of health-promoting behaviors among nursing students was 2.55±0.35. Hong et al. (2007) reported the mean and standard deviation of health-promoting behaviors among nursing students to be 2.99±0.33. McElligott et al. at 2010 estimated the mean of health-promoting behaviors among nurses to be 2.60±0.41. The findings of the present study correspond to findings of McElligott et al. (2010) but are lower than findings of Hung et al. (2007).

In the present study, subscale of physical activity has the lowest mean and subscale of interpersonal relations has the highest mean. McElligott et al. at 2010 found that stress management has the lowest mean and interpersonal relationship have the highest mean (McElligott et al., 2010). The findings of these two studies differ in highest and lowest means. The difference between findings may lie in cultural issues, limitation of sport equipment and facilities in Iran, lack of time management, lack of good policies for leisure time, and lack of attention by people and authorities to adverse consequences of immobility. In a study on students of Yazd city, the highest score belonged to subscale of spiritual growth and the lowest score belonged to subscale of physical activity (Motlaq et al., 2010). These findings correspond to our findings.

In the present study, subscales of nutrition, interpersonal relationship, and spiritual growth of students had high scores, but other subscales of Health-Promoting Lifestyle Profile 2 had fair scores. In this connection, Hensel reported that four subscales of health-promoting behaviors among nurses had fair scores (2.5–2.9) which indicated that health-promoting behaviors of nurses need to be improved (Hensel, 2011).

In the present study, score quarters of health-promoting behaviors showed that the majority of students had perceived health-promoting behaviors at a high level. Unlike our findings, Hong et al. reported total score of health-promoting behaviors among students to be at a fair level (Hong et al., 2007). Likewise, Soleimani et al. (2005) reported health-promoting behaviors among students to be at fair level. In their study, only 12.7% of first year nursing students had good health behaviors with respect to the factors associated with coronary artery diseases (Soleimani et al., 2005). The difference between our findings and those of Solimani et al. (2005) may lie in the population under study and the focus on a specific disease in their research. Also, the difference between our findings and those of Hung et al. may lie in cultural differences.

In the present study, the mean of nutrition, physical activity, health responsibility, and stress management among the majority of students was at a fair level, and mean of interpersonal relationship and spiritual growth was at a high level. In a research on students of Tehran University of Medical Sciences, nutritional behaviors of medicine and non-medicine students was reported to be at a good level (Shaban et al., 2007). These findings do not correspond to our findings. The reason for this difference may lie in the fact that samples were in a different situation. Hong et al. found that interpersonal relations had the highest score (Hong et al., 2007), which corresponds to our findings.

According to our findings, the mean of health-promoting behaviors among female students was higher than among male students, but the difference was not statistically significant. Hong et al., too, found that there was no significant difference between male and female nursing students in health-promoting behaviors (Hong et al., 2007). In the study made by Hawks et al. on health-promoting behaviors among American and Japanese students, the mean of female students were significantly higher than those of men, but the difference between Japanese female and male students was not significant (Hawks et al., 2002). Their findings on Japanese students correspond to our findings, but their findings on American students do not correspond to our findings. The reason for the difference may lie in the difference between western and eastern cultures.

According to our findings, the mean of female students in subscales of nutrition, interpersonal relationship, health responsibility, spiritual growth, and stress management were higher than male students. Male students had better score in subscales of nutrition and physical activity. In subscales of nutrition, physical activity, interpersonal relationship, and health responsibility, the difference was significant. According to the findings of Larouche (1998) on 151 students, female students showed a significantly better behavior than male students in nutrition, interpersonal relationship, and health responsibility (Larouche, 1998), which corresponds to our findings. In this connection, the findings of Deenan on Thai Adolescents showed that men participated in physical activities (sport) more than women did (Deenan, 2003). The findings of Hong et al. (2007), and Choi Hui (2002), unlike our findings, showed that there is no statistically significant difference between two genders in subscales of health-promoting behaviors. The difference between our findings and the above said studies in subscale of physical activity may lie in access to sports facilities and the freedom of male students compared to female students in Iran. In Turkey, Daskapan et al. (2006) found that the regular physical activity among female students was lower than male students, which corresponds to our findings (Daskapan et al., 2006).

The findings of this study showed that the means of physical activity and stress management among single students are more than among married students. In other subscales, married students had higher scores than single students. Independent T test showed that there is a significant difference between single and married students in subscales of nutrition and spiritual growth (p<0.05). According to a study on American students, married students had a significantly higher score in subscale of health responsibility (Stark et al., 2005). The findings of this study correspond to our findings with respect to higher score of married students, but the reason why the difference is not significant in our findings may lie in the low number of married student.

According to our findings, the mean of health-promoting behaviors among first and third year students was higher than among students of other years, but the difference was not statistically significant. In this connection, Hong et al. found that health-promoting behaviors among students of last years were significantly better (Hong et al., 2007). Alpar et al. 2008, too, found a significant difference between newly-admitted nursing students and students of last year in these behaviors (Alpar et al., 2008). According to the findings of Wittayapun et al. (2010) on 323 nursing students in Thailand, fourth year students had a significantly higher score than first and third year students (Wittayapun et al., 2010), These findings do not correspond to our findings. In the present study, study in nursing discipline was not found to be effective in health promotion among students. On the other hand, students of different academic years perceived health-promoting behaviors at a high level. In this respect, we can conclude that they had such behaviors in their whole life. Moreover, scores of students of upper years increased in certain subscales of health-promoting behaviors (health responsibility) and reduced in some others (stress management).

In the present study, the mean of stress management among first year students was higher than that of other students. On the other hand, fourth year students perceived health responsibility more than other students, which was a significant difference. Likewise, Alpar et al. (2008) found that there was a statistically significant difference between students of first years and students of last years in terms of health responsibility (Alpar et al., 2008). The reason may lie in entrusting the nursing students with responsibility of patients in their last academic year. In this connection, Alpar et al. (2008) found that all subscales of health-promoting behaviors among last year students were higher than newly-admitted students (Alpar et al., 2008). Hong et al. at 2007 found that the score of health responsibility and stress management among first year students was lower than that of other students (Hong et al., 2007). In the present study, however, the mean of subscale of stress management among first year students was higher than that of last year students, which was a statistically significant difference. The reason for difference between our findings and those of Alpar et al. (2008) and Hong et al. (2007) with respect to stress management may lie in stressful and competitive atmosphere of nursing in Iran. In addition, educational conditions in Iran may be more stressful than in Turkey. In this matter, stress reduction solutions seem to be among the priorities of nursing discipline.

In the present study, the mean of nutrition and physical activity among first year students was higher than last year students. This score reduction may indicate that students acquire theoretical knowledge but fail to put it into practice due to various reasons such as living in dormitory, pressure of high volume of courses, long distances, and long working hours in hospital. In this respect, Hensel points out that there has been an association between inefficient stress management among nurses and their reduced participation in other health-promoting behaviors (Hensel, 2011).

In the present study, the mean of health-promoting behaviors in low body mass index group was higher than other groups. Tukey test indicated a significant difference in fat group (p>0.05). Hong et al. at 2007 found no significant difference between different body mass indexes in terms of health-promoting behaviors (Hong et al., 2007). The findings of the present study with respect to health-promoting behaviors are contrary to other findings, as people with normal body mass index seem to have better score or at least equal score. In this matter, Deenan (2003) found that there is a relationship between lower body mass index and higher levels of exercise among Thai adolescents (Deenan et al., 2003).

## 5. Conclusion

This study analyzed health-promoting behaviors among nursing students. Considering that this is the first quantitative study to be made on health promotion among nursing students in Iran, the findings of this study can help policymakers, managers and instructors to plan and execute practical programs for promoting the health level among nursing students.

## References

[ref1] Alpar S. E, Senturan L, Karabasak U, Sabuncu N (2008). Change in the health promoting lifestyle behavior of Turkish University students from beginning to end of nurse training. Nurse Education in Practice.

[ref2] Canadian Nursing Students’ Association (2009). Self-Care Practices among Nursing Students. Nursing: Change, Challenge, Choice. http://aeic.ca/english/publications/policies-and-position-statements/position-statements/self-care-practices-among-nursing-students.

[ref3] Chhim D (2011). Comparison of Health Promotion lifestyles of Family Nurse Practitioner Students and Non-Nursing Students. A Thesis presented to school of Nursing. California State University, Long Beach. In Partial Fulfillment of the Requirements for the Degree Master of Science in Nursing.

[ref4] Choi Hui W-H (2002). The health-promoting lifestyles of undergraduate nurses in Hong Kong. Journal of Professional Nursing.

[ref5] Daskapan A, Handan Tuzun E, Eker L (2006). Perceived barrier to physical activity in university students. Journal of Sport Science and Medicine.

[ref6] Deenan A (2003). Testing the health promotion model with Thai adolescents. A dissertation presented to the faculty of the Grade School of Saint Louis University in Partial fulfillment of the Requirements for the Degree of Philosophy. ProQuest Dissertations and Theses; ProQuest pg. n/a.

[ref7] Eshah N. F (2011). Lifestyle and health promoting behaviours in Jordanian subjects without prior history of coronary heart disease. International Journal of Nursing Practice.

[ref8] Haddad L, Kane D, Rajacich D, Cameron Sh, Al-Ma’aitah R (2004). A Comparison of Health Practices of Canadian and Jordanian Nursing Students. Public Health Nursing.

[ref9] Hawks S. R, Madanat H. N, Merrill R. M, Goudy M. B, Miyagawa T (2002). A Cross-cultural Comparison of Health Promoting Behaviors among College Students. The International Electronic Journal of Health Education.

[ref10] Hensel D (2011). Relationships among nurses’ professional self concept, health, and lifestyles. Western Journal of nursing Research.

[ref11] Hong F. J, Sermesri S, Keiwkarnka B (2007). Health-promoting lifestyles of nursing students in Mahidol University. Journal of Public Health and Development.

[ref12] Hosseini M, Yaghmaei F, Hosseinzadeh S, Alavi Majd H (2010). Translation and psychometric properties of the Persian version of the health promotion lifestyle profile 2. School of Nursing and Midwifery Research Project. Shahid Beheshti University of Medical Sciences and Health Services 2010.

[ref13] Information Center of Iran Medical Council (2011). For the first time national code of ethics of the nursing profession was announced. 2011. http://ino.ir/tabid/40/ctl/ArticleView/mid/384/articleId/1074/language/fa-IR/----.aspx.

[ref14] Irazusta A, Gil S, Ruiz F, Gondra J, Jauregi A, Irazusta J (2006). Exercise, physical fitness, and dietary habits of first year nursing students. Biological Research for Nursing.

[ref15] Jackson E. S, Tucker C. M, Herman K. C (2007). Health value, perceived social support, and health self- efficacy as factors in a health – promoting lifestyle. Journal of American College Health.

[ref16] Larouche R (1998). Determinants of college students’ health promoting lifestyle. Clinical Excellence for Nurse Practitioners.

[ref17] Liimatainen L, Poskiparta M, Sjogren A, Kettunen T, Karhila P (2001). Investigating student nurses’ constructions of health promotion in nursing education. Health Education Research.

[ref18] McElligott D, Capitulo K. L, Morris D. L, Click E. R (2010). The effect of a holistic program on health – promoting behaviors in hospital registered nurses. Journal of Holistic Nursing.

[ref19] Motlaq Z, Mazloomi Mahmoodabad S. S, Momayyezi M (2010). Health Promotion Behaviours in Students. Zahedan Journal for Medical Sciences Researches (Tabibe Sharq).

[ref20] Peterson S. J, Bredow T. S (2004). Middle Ranged Theories: Application to Nursing Research.

[ref21] Shaban M, Mehran A, Taghlili F (2007). The relationship between health perception and health behaviors compared two groups of fourth year students at Tehran University and Tehran University of Medical Sciences. Hayat.

[ref22] Soleimani A, Taghlili F, Rasouli M (2005). Health behaviors of nursing freshmen in relation to risk factors for coronary artery disease. Journal of Nursing and Midwifery, Hamedan.

[ref23] Stark M. A, Manning-Walsh J, Vliem S (2005). Caring for self while learning to care for others: A challenge for nursing students. Journal of Nursing Education.

[ref24] Walker S. N, Hill-Polerecky D. M (1996). Psychometric evaluation of the Health Promoting lifestyle profile 2. Unpublished manuscript, University of Nebraska Medical Centre.

[ref25] Wittayapun Y, Tanasirirug V, Butsripoom B, Ekpanyaskul C (2010). Factors affecting health- promoting behaviors in nursing students of the Faculty of Nursing, Srinakharinwirot University, Thailand. Journal of Public Health.

